# Long-term treatment with metformin in the prevention of fatty liver in Zucker diabetic fatty rats

**DOI:** 10.1186/s13098-019-0491-1

**Published:** 2019-11-12

**Authors:** Yi Sui, Xianhe Kong, Rongrong Fan, Yanbin Ye, Haiyan Mai, Shuyu Zhuo, Wei Lu, Peishan Ruan, Shi Fang, Tao Yang

**Affiliations:** 1 Department of Clinical Nutrition, The First Affiliated Hospital, Sun Yat-sen University, Guangzhou, 510080 China; 2 Endoscopy Center, The Sixth Affiliated Hospital, Sun Yat-sen University, Guangzhou, 510655 China; 3 Department of Biosciences and Nutrition, Karolinska Institute, Stockholm, Sweden; 4 Center for Medical Research and Innovation, Shanghai Pudong Hospital, Fudan University Pudong Medical Center, Shanghai, 201399 China

**Keywords:** Metformin, Non-alcoholic fatty liver disease, Zucker diabetic fatty rats, Gene expression profiling

## Abstract

**Background:**

Treatment with metformin, the biguanide of hepatic insulin sensitizer, in patients with non-alcoholic fatty liver disease (NAFLD) has been reported with contradictory findings regarding the effectiveness on blood lipids and liver histology. In this study, we aimed to explore the preventive effects of metformin on NAFLD in Zucker diabetic fatty (ZDF) rats.

**Methods:**

Male ZDF rats and Zucker lean rats aged 4–8 weeks were subjected to vehicle or metformin treatment for 6 months. Liver cDNA microarray assay, and protein semiquantitative and histological examinations were performed.

**Results:**

Data demonstrated that ZDF rats developed hyperglycemia, hyperlipidemia, liver deficiency and hepatocyte degeneration. The metformin treatment significantly reduced post-load blood glucose levels, but not blood lipid profiles or liver enzyme levels. Hepatocyte degeneration was not attenuated after the treatment. The metformin-treated ZDF rats showed activation of AMP-activated protein kinase by Western blot and overexpression of cytochrome c oxidase by immunofluorescent microscopy. Gene expression microarray assay demonstrated that a panel of genes participating in glucose and lipid metabolisms were changed in the ZDF rats, and most of the altered genes involved in glucose and cholesterol metabolisms, but not those in fatty acid metabolisms, were corrected by the metformin treatment. No genes associated with inflammation, apoptosis, fibrosis, or cell death were overexpressed in the metformin-treated ZDF rats.

**Conclusions:**

These results suggest that long-term metformin treatment presents no preventive effect for NAFLD in ZDF rats.

## Background

Metformin, a biguanide that improves hepatic insulin resistance and hyperglycemia, is the first-line therapeutic agent for patients with type 2 diabetes, especially those who also have hyperlipidemia and obesity [1, 2]. Since it was noted that some patients with fatty liver diseases had improved liver function tests after metformin treatment, metformin was studied as a candidate for the treatment of non-alcoholic fatty liver disease (NAFLD) [3–6]. Short-term treatment with metformin has frequently been reported with beneficial effects on lowering blood lipid levels and protecting hepatocytes from lipid accumulation [2, 7–9], but several studies with long-term metformin treatment did not show hepatic histological protection [6, 10–12]. Paradoxically, metformin might induce hepatotoxicity including acute hepatitis [13–16] and cholestasis [17]. In all the cases, several weeks of metformin treatment induced elevated hepatic enzyme concentrations. These adverse effects resolved after discontinuation of metformin treatment. The mechanisms underlying the lipid-lowering effects and hepatotoxicity are still unclear.

The primary purpose of present study was to test the efficacy of metformin on the prevention of fatty liver disease in Zucker diabetic fatty (ZDF) rats. Adult ZDF rats develop hyperlipidemia, hyperglycemia, hyperinsulinemia, insulin resistance, fatty liver and hepatocyte degeneration owing to an inherited mutation of the leptin receptor. Hereby, young ZDF rats were treated with metformin for 6 months, followed by mRNA liver tissue array to investigate the long-term effects of metformin treatment on the development of hyperlipidemia and fatty liver disease.

## Materials and methods

### Rat experiments

Zucker fatty rats were obtained from Beijing Vital River Laboratory Animal Co., Ltd. (Beijing, China) and introduced into the Laboratory Animal Services Centre at the Fudan University Pudong Medical Center. The animals were caged in pairs, housed at 23 ± 1 °C with a 12-h dark/light cycle, having free access to water and fed on a standard laboratory rat diet (5001 Rodent Diet, LabDiet, St Louis, MO). Ethical approval for animal studies was according to the Animal Experimentation Ethics Committee of Fudan University Pudong Medical Center.

Fourteen male ZDF rats and six Zucker lean rats aged 4–8 weeks were divided into three groups: (1) ZDF rats treated with metformin (50 mg/kg body weight, n = 7), (2) ZDF rats treated with vehicle of distilled water (10 mL/kg, *n* = 7), and (3) Zucker lean rats treated with vehicle of distilled water (10 mL/kg, *n* = 6). All treatments were conducted through oral administration once daily. After the 6-month treatment, rats were killed at fasting state. Liver tissues were collected freshly for cDNA microarray or fixed in 10% neutral formaldehyde for histological analysis.

Body weight was monitored before rats were killed; oral glucose tolerance tests (OGTT) were conducted by gavage of dextrose (2.5 g/kg body weight) following 8 h of fasting. Blood from the tail vein was used for measuring blood glucose levels with a blood glucose meter (Onetouch Ultra, LifeScan, Inc.; US). Fasting blood samples were taken for the measurement of blood lipids and liver functions.

### Total RNA isolation and reverse transcription

Total RNA from rat livers was extracted using RNeasy Mini Kit with DNase digestion option (Qiagen, Valencia, CA) to remove residual genomic DNA according to the manufacturer’s instructions. Total RNA prepared from individual animals was pooled together using equal amount of total RNA from individual animals in each group. The total RNA samples were sent to Li Ka Shing Institute of Health Sciences Core Lab (The Chinese University of Hong Kong, Hong Kong) for reverse transcription, labeling, microarray hybridization, washing, and scanning using the Affymetrix GeneChip^®^ Gene 1.0 ST Array System. Briefly, 100 ng of the pooled total RNA was used as a template for cDNA synthesis and subsequent labeling using the GeneChip^®^ Whole Transcript Sense Target Labeling Assay from Affymetrix (Affymetrix, Santa Clara, CA). The generation of hybridization cocktails, hybridization to DNA microarrays, and fluorescent labeling of RNA targets were carried out as described in the standard Affymetrix protocol. Fluorescence-labeled microarrays were scanned with GeneChip^®^ Scanner 3000 (Affymetrix). Results were analyzed with GeneSpring GX 10.0 software with global normalization and fold change ≥ 2.0 was chosen as the cut-off of different expression levels of the compared genes.

### Real-time quantitative PCR

The mRNA samples were estimated via quantification of relative expression levels using TaqMan 2X Universal PCR Master Mix (Applied Biosystems Inc, CA) according to the manufacturer’s protocols. For mRNA amplification, primers were synthesized by Invitrogen (Invitrogen, Cergy-Pontoise, France) as listed in Table 1. Real-time PCR was performed under the following conditions: 95 °C, 10 min; 95 °C, 15 s followed by 60 °C, 60 s and 40 times of cycles. PCR amplifications were performed on a 7900HT Fast real-time PCR system and data were analyzed with the 7900HT Fast system SDS software v2.0 (Applied Biosystems). Relative mRNA expression was quantified by the comparative Ct method and expressed as 2^− ΔΔCt^.

**Table 1:** Primer sequences for real-time PCR

Gene name	Primer sequences	
G6pdx	Forward	5′-GAC TGT GGG CAA GCT CCT CAA-3′
	Reverse	5′-GCT AGT GTG GCT ATG GGC AGG T-3′
Hmgcs1	Forward	5′-TCA AGG CTT GAC TCA AGA ACG-3′
	Reverse	5′-GGA ATA TGC TCT GTA GCT GTG-3′
Cyp7a1	Forward	5′-ATG ACA CGC TCT CCA CCT TTG A-3′
	Reverse	5′-AGC TCT TGG CCA GCA CTC TGT-3′
Igfbp1	Forward	5′-GAA GCT TTT CTC ATC TCC ATA CAT GT-3′
	Reverse	5′-AAG GCC CCT ACC TCA GAC TGA-3′
Scd-1	Forward	5′-GCT TGT GGA GCC ACA GGA CT-3′
	Reverse	5′-ATC CCG GGC CCA TTC ATA-3′

### Western blot

Total protein extracts were prepared from frozen liver tissues. Briefly, tissue was homogenized in a RIPA buffer and protein extracts (100 μg) were separated by SDS-PAGE using 10% gels. The resolved proteins were then transferred onto nitrocellulose membranes and incubated with primary antibodies of rabbit anti-AMPKα (1:1000, Cell Signaling Technology, Danvers, MA), rabbit anti-p-AMPK (1:1000, Cell Signaling Technology), and rabbit anti-β-actin (1:10,000, Abcam, Cambridge, MA) overnight at 4 °C. After washing with TBS-T, membranes were incubated with anti-rabbit secondary antibody conjugated to horseradish peroxidase (Upstate, Temecula, MA) with dilution of 1:5000. Protein bands were detected by enhanced chemiluminescence reagent (Amersham, Piscataway, NJ) and then exposed to Hyperfilm. The major protein bands detected were approximately 62 KD for AMPK and p-AMPK, 47 KD for β-actin. Signals were then quantitated by densitometry and corrected for the β-actin signal.

### Histopathological examination

Liver tissues fixed in neutral formaldehyde were embedded in paraffin. Sections (4 μm) were stained with periodic acid–Schiff (PAS) for routine structural examination. For immunofluorescence examination, slides were stained with antibody of mouse anti-cytochrome c oxidase (CCO, 1:200, Invitrogen, Cergy-Pontoise, France) to evaluate the mitochondria function and counterstained with DAPI. Stained slides were examined with a Zeiss Axioplan 2 imaging microscope (Carl Zeiss, Hamburg, Germany), and representative images were captured using a SPOT digital camera (Diagnostic Instruments Inc, Sterling Heights, MI).

### Statistical analysis

Data are expressed as mean ± standard deviation. SPSS 16.0 (Statistics Package for the Social Sciences 16.0, Chicago, IL) was used to perform statistical analysis. One-way ANOVA was adopted to analyze the statistical differences of biochemical parameters among all groups, and Bonferroni test was used for post hoc multiple comparisons between different groups. A two-tailed *p* value of less than 0.05 was taken as the criterion for a statistically significant difference.

## Results

### Biochemical and metabolic profiles

During this study of 6 months, the ZDF rats showed progressive body weight gain compared with age-matched Zucker lean rats (Table 2). The weight gain in ZDF rats was not affected by the 6-month metformin treatment. Metformin did not significantly change blood total cholesterol and triglyceride in ZDF rats. However, treatment with metformin indeed significantly reduced the 2-h OGTT blood glucose levels.

**Table 2:** Metabolic characters of rats after 6-month treatment

	Zucker lean, *n* = 6	ZDF + Met, *n* = 7	ZDF + V, *n* = 7
Body weight (g)	390 ± 12	491 ± 29^a^	485 ± 49^b^
2-h OGTT blood glucose (mmol/L)	7.9 ± 1.7	11.4 ± 2.3^ac^	14.7 ± 2.3^b^
Serum total cholesterol (mmol/L)	1.07 ± 0.07	4.9 ± 1.0^a^	5.0 ± 0.6^b^
Serum triglyceride (mmol/L)	0.32 ± 0.02	8.9 ± 3.3^a^	8.7 ± 1.9^b^
Serum total protein (g/L)	70.0 ± 1.7	70.6 ± 1.7	73.0 ± 2.3
Serum albumin (g/L)	20.7 ± 0.6	16.1 ± 2.0^a^	15.7 ± 1.4^b^
ALT (U/L)	79.3 ± 11.4	139.9 ± 25.9^a^	155.7 ± 79.2^b^
AST (U/L)	86.7 ± 6.1	120.6 ± 22.8^a^	156.3 ± 61.0^b^

Data are mean ± SD

*OGTT* oral glucose tolerance test, *ALT* alanine aminotransferase, *AST* aspartate transaminase

^a,b^*p *< 0.05 versus Zucker lean, ^c^ *p *< 0.05 versus V

ZDF rats developed liver deficiency at the age of 8 months after the 6-month vehicle treatment, as reflected by decreased serum albumin level and elevated serum alanine aminotransferase (ALT) and aspartate transaminase (AST) levels. Compared with the vehicle, metformin treatment showed a trend of moderating the increase of liver enzyme levels, but this trend was not statistically significant (Table 2).

### Histological assessment of liver

**Fig. 1:**
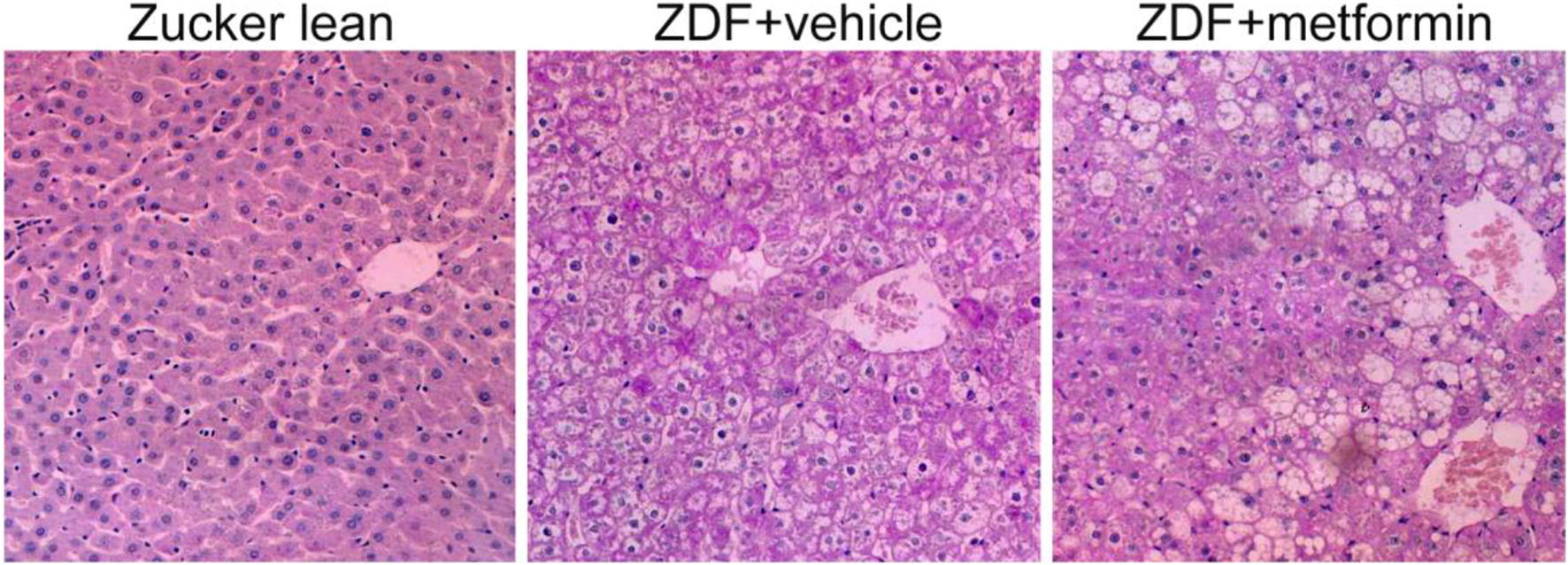
Ballooning degeneration and fatty change of hepatocytes in Zucker rats. Liver tissues were collected after 6-month treatment with vehicle or metformin. PAS staining demonstrated patch-distributed ballooning degeneration and fatty change of hepatocytes in vehicle- and metformin-treated ZDF rats

PAS staining and light microscopy were performed to illustrate the morphological changes of liver histology. The untreated ZDF rats showed patches of ballooning degeneration and fatty change with cytoplasmic microvacuolation in hepatocytes (Fig. 1). These changes were even more obvious in metformin-treated ZDF rats, in which patches of the degenerated cells were surrounded with condensed hepatocytes.

### AMPK activation and cytochrome c oxidase (CCO) overexpression

**Fig. 2:**
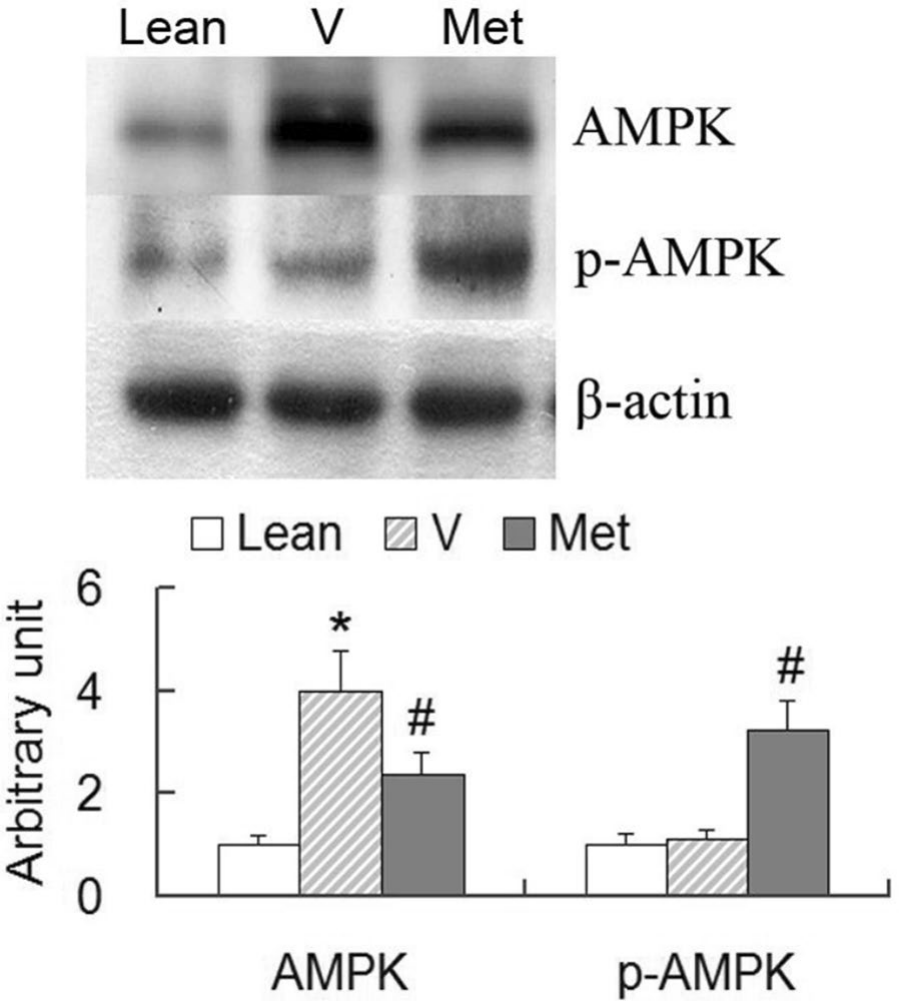
Protein expression of liver AMPK. Liver tissues were obtained from the Zucker lean rats (ZL), vehicle-treated ZDF rats (V) and metformin-treated ZDF rats (Met) after 6-month treatment. Equal amounts of liver lysates (100 μg) were subjected to SDS-PAGE to immunoblot for AMPK and phosphorylated-AMPK (p-AMPK). Metformin-treated rats showed activation of AMPK. Data are mean ± SD, *p < 0.05, V versus ZL; ^#^*p *< 0.05, Met versus V

Based on the previous study which demonstrated metformin lowered blood glucose and lipids by activating AMP-activated protein kinase (AMPK) [18], protein expression level of AMPK was semi-quantitatively measured by Western blot. Increased protein levels of total AMPK but not phosphorylated-AMPK (p-AMPK) were found in vehicle-treated rats compared with that in Zucker lean rats. Metformin treatment increased p-AMPK but not the total AMPK protein level compared with those in vehicle-treated rats (Fig. 2). The higher ratio of p-AMPK to total AMPK level implied the activation of AMPK in metformin-treated ZDF rats.

**Fig. 3:**
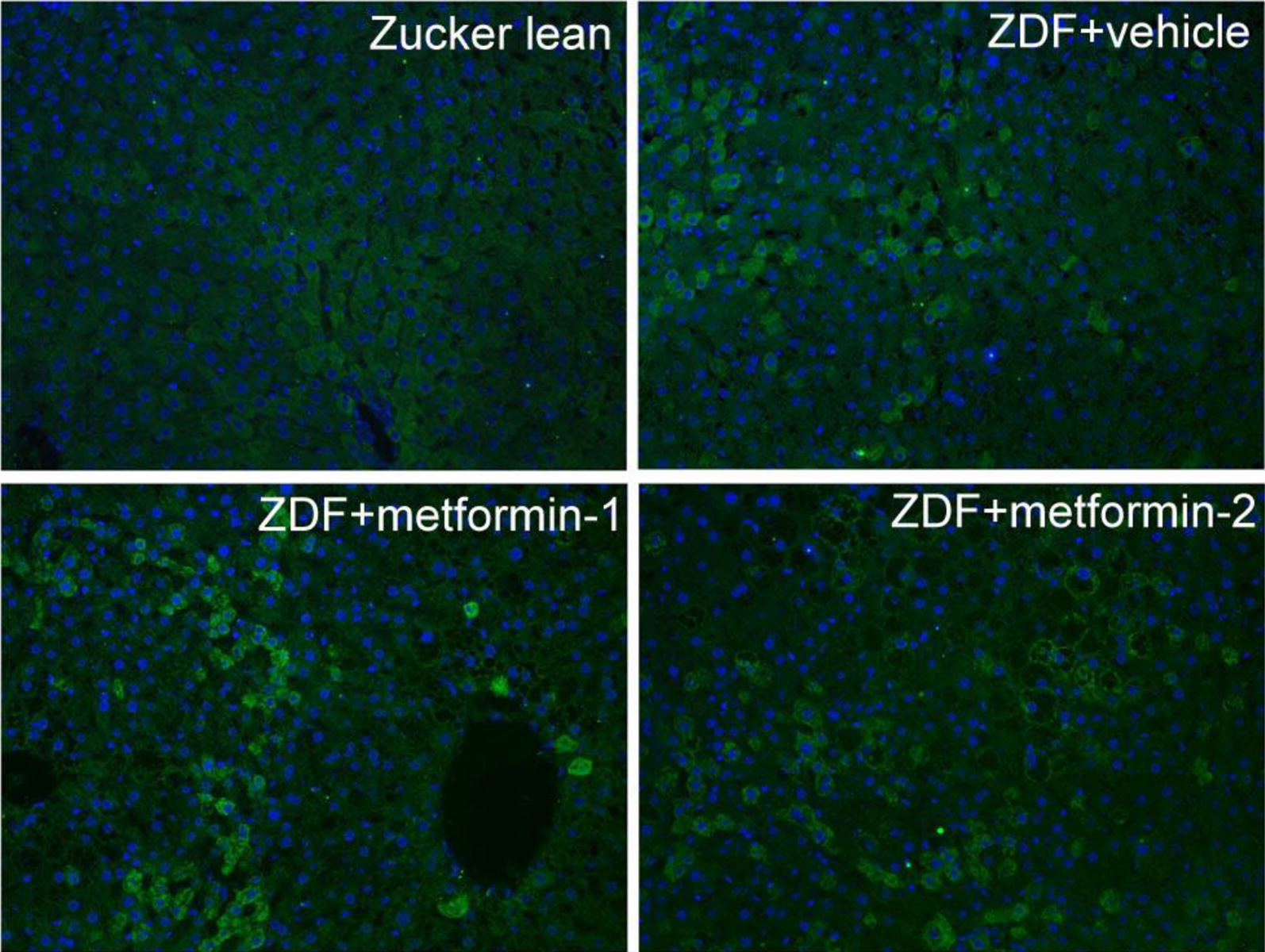
Immunofluorescence microscopy of cytochrome c oxidase (CCO). Liver tissues obtained after 6-month treatment with vehicle or metformin were stained with anti-CCO (green). The CCO staining was weak and uniform in Zucker lean rats, whereas Zucker diabetic fatty rats showed patched of strong CCO reactivity. Stronger CCO labeling was seen at the intracellular vacuoles in hepatocytes with ballooning degeneration from metformin-treated rats (ZDF + metformin-1, (ZDF + metformin-2)

Considering the important roles of CCO in the processes of glucose and lipid catabolisms in mitochondria, protein expression level of CCO was analyzed by immunofluorescence microscopy. There was a homogeneously weak staining of CCO in the normal liver of Zucker lean rats (Fig. 3). The vehicle-treated ZDF rats showed scattered or stripe-like distribution of positive labeling, while the metformin-treated rats demonstrated patches of strong cytoplasmic staining of CCO. In metformin-treated rats, the positively stained hepatocytes were usually found adjacent to degenerated hepatocytes which often exhibited weak positivity. Occasionally, edges of the cytoplasmic vacuoles were strongly stained with CCO antibody.

### Global profiling of mRNA expression after metformin treatment

The activity of hepatocyte in substrate catabolic processes as reflected by AMPK activation and CCO overexpression in the metformin-treated ZDF rats was not consistent with the observation that metformin did not attenuate blood lipids and fatty liver. To elucidate the impact of long-term metformin treatment on global gene expressions, we examined the profiling of mRNA expression levels using microarray technology. Microarray analysis detected 22,342 liver genes in total, while metformin treatment altered the expression levels of 92 genes on the criteria of fold change ≥ 2.0 when compared with those of the untreated ZDF rats. Among the 92 genes, 39 genes (42.4%) were involved in metabolic processes, including 16 genes in lipid metabolism, 11 in sterol/steroid metabolism, 3 in fatty acid metabolism, 7 in glucose metabolism, and 4 genes in bile acid metabolism.

We further examined the genes amended by the metformin treatment. In all the detected genes, 66 genes were significantly altered by the gene mutation of leptin receptor in the ZDF rats compared with Zucker lean rats on the criteria of fold change ≥ 2.0 (Table 3). In the annotated 55 genes, most of them were related to the metabolic processes, including 12 genes in lipid metabolism, 5 in sterol/steroid metabolism, 4 in fatty acid metabolism, 4 in glucose metabolism, and 3 in bile acid metabolism. However, none of the genes in insulin signaling pathway such as insulin receptor, insulin receptor substrates and glucose transporters were affected by the leptin receptor mutation in ZDF rats even when we reduced the fold change criterion to 1.5. Among the 55 significantly altered genes, 36 (65%) genes were corrected by metformin treatment (Table 3), including 6 (50%) genes in lipid metabolism, 4 (80%) in sterol/steroid metabolism, 3 (75%) genes in glucose metabolism, and 2 (67%) genes in bile acid metabolism. Remarkably, all the six corrected genes in lipid metabolisms were related to cholesterol metabolism, while none of the genes were related to fatty acid metabolism (Table 3).

**Table 3:** Genes modified by metformin treatment

Gene symbol	V vs. ZL		Met vs. V		Gene description
*Genes corrected by metformin*					
Igfbp1^g^	5.25	↓	19.28	↑	Insulin-like growth factor-binding protein 1
Ccrn4lb	4.03	↓	10.24	↑	CCR4 carbon catabolite repression 4-like B
Stac3	12.77	↓	9.35	↑	SH3- and cysteine-rich domain 3
Sds^g^	4.83	↓	8.56	↑	Serine dehydratase
Pnpla3	13.03	↑	8.23	↓	Patatin-like phospholipase domain containing 3
Lcn2	19.50	↑	7.81	↓	Lipocalin 2
Btg2	2.09	↓	6.96	↑	B-cell translocation gene 2, anti-proliferative
Coq10b	2.34	↓	5.41	↑	Coenzyme Q10 homolog B
A2m	6.91	↑	5.40	↓	Alpha-2-macroglobulin
Cyp7a1^bcs^	2.81	↓	5.01	↑	Cytochrome P450, family 7, subfamily a, polypeptide 1
Cdh17	8.39	↓	4.66	↑	Cadherin 17
LOC313220^b^	5.07	↓	4.21	↑	Similar to bile acid coenzyme A
Sqle^s^	2.25	↑	4.13	↓	Squalene epoxidase
Me1	2.55	↑	4.12	↓	Malic enzyme 1, NADP(+) dependent, cytosolic
G6pdx^g^	3.01	↑	3.81	↓	Glucose-6-phosphate dehydrogenase X-linked
Rhbdd2	2.51	↓	3.77	↑	Rhomboid domain containing 2
Cml4	4.49	↓	3.48	↑	Camello-like 4
Car3	4.71	↓	3.30	↑	Carbonic anhydrase 3
Inmt	8.28	↓	3.28	↑	Indolethylamine *N*-methyltransferase
Nrep	2.78	↓	3.08	↑	Neuronal regeneration-related protein
Hmgcs1^cs^	2.00	↑	3.07	↓	3-Hydroxy-3-methylglutaryl-coenzyme A synthase 1
Mup5	3.18	↓	3.05	↑	Major urinary protein 5
RGD1565709	2.38	↓	3.03	↑	Similar to ovostatin-2
RGD1562060	3.43	↑	2.92	↓	Similar to short-chain dehydrogenase reductase 9
Ddhd1	2.84	↑	2.89	↓	DDHD domain containing 1
Por	2.19	↓	2.78	↑	P450 (cytochrome) oxidoreductase
Cyp3a9^s^	3.55	↓	2.69	↑	Cytochrome P450, family 3, subfamily a, polypeptide 9
Slc1a2	2.64	↓	2.46	↑	Solute carrier family 1, member 2
Fmo1	3.91	↓	2.45	↑	Flavin-containing monooxygenase 1
Dhrs7	3.59	↓	2.39	↑	Dehydrogenase/reductase (SDR family) member 7
Aox3	3.22	↓	2.27	↑	Aldehyde oxidase 3
LOC360228	4.29	↑	2.25	↓	WDNM1 homolog
Rdh2	2.99	↓	2.17	↑	Retinol dehydrogenase 2
Hao2	2.73	↓	2.13	↑	Hydroxyacid oxidase 2 (long chain)
Nox4	3.57	↓	2.07	↑	NADPH oxidase 4
Obp3/Mup4	2.16	↓	2.00	↑	Alpha-2u globulin PGCL4/major urinary protein 4
*Genes uncorrected by metformin*					
Dbp	2.35	↓	1.50	↑	D site albumin promoter-binding protein
Acsm3^f^	2.72	↓	1.82	↑	Acyl-CoA synthetase medium-chain family member3
Chka	2.06	↑	1.80	↓	Choline kinase alpha
Scd1^f^	2.02	↑	1.47	↓	Stearoyl-coenzyme A desaturase 1
Ugp2^g^	2.16	↓	1.23	↑	UDP-glucose pyrophosphorylase 2
Inhba	2.25	↑	1.56	↓	Inhibin beta-A
Prtfdc1	2.17	↓	1.61	↑	Phosphoribosyl transferase domain containing 1
Es22	2.00	↓	1.30	↑	Esterase 22
Ces3	2.66	↓	1.52	↑	Carboxylesterase 3
Esm1	2.20	↑	1.74	↓	Endothelial cell-specific molecule 1
Prlr	2.53	↑	1.63	↓	Prolactin receptor
Fabp7^f^	2.14	↓	1.63	↑	Fatty acid-binding protein 7, brain
Slc16a10	2.10	↓	1.77	↑	Solute carrier family 16, member 10
Upp2	3.33	↓	1.95	↑	Uridine phosphorylase 2
Insig1^cs^	3.06	↑	1.54	↓	Insulin-induced gene 1
RGD1561619	2.83	↓	1.79	↑	Similar to Camello-like 2
Slco1a4	2.20	↓	1.23	↑	Solute carrier organic anion transporter family, member 1a4
Adfp^f^	2.06	↑	1.25	↓	Adipose differentiation-related protein
Ces3	2.50	↓	1.62	↑	Carboxylesterase 3

^b^Bile acid metabolisms, ^c^ cholesterol metabolisms, ^f^ fatty acid metabolisms, ^g^ glucose metabolisms, ^s^ sterol metabolisms

In the amended genes, the most significantly altered gene is insulin-like growth factor-binding protein-1 (IGFBP1), a growth-related gene. IGFBP1 was down-regulated by 5.3-fold by the leptin receptor gene mutation in the vehicle-treated ZDF rats and up-regulated by 19.3-fold by metformin treatment. Another important gene corrected by the metformin treatment was Cyp7a1 (cytochrome P450, family 7, subfamily a, polypeptide 1), the key enzyme in bile acid synthetic process for the removal of cholesterol from the liver in the form of bile acid. As shown in Table 3, the metformin treatment also corrected other genes encoding the key enzymes in multiple metabolic processes, such as glucose-6-phosphate dehydrogenase (G6pdx, the rate-limiting enzyme of the pentose phosphate pathway in maintenance of NADPH), and HMG-CoA synthase 1 (Hmgcs1, an enzyme in cholesterol synthesis).

Not all the genes altered by the leptin receptor gene mutation were corrected by the metformin treatment. Among the 55 genes altered in vehicle-treated ZDF rats compared with Zucker lean rats, 19 (35%) genes remained uncorrected in the metformin-treated ZDF rats. These included five genes involved in lipid metabolism (predominantly in fatty acid metabolism), one gene in glucose metabolism, and one gene in bile acid metabolism (Table 3).

Since degeneration and steatosis in hepatocytes were observed in metformin-treated ZDF rats, we examined the expression alterations of genes associated with possible cell damages such as inflammation, cell death, apoptosis, cell growth, fibrosis, and genes responsible for stresses (oxidative stress and endoplasmic reticulum stress). In the 38 genes which reached the criteria of fold change ≥ 2.0 in the metformin-treated ZDF rats compared with Zucker lean rats, 23 genes (60.5%) were annotated, among which, 1 gene was involved in cell cycle (Snf1lk, SNF1-like kinase), and 2 in cell growth (Igfbp1, and Socs2, namely suppressor of cytokine signaling 2). Moreover, no gene correlated with inflammation, cell death, apoptosis, fibrosis, oxidative stress or endoplasmic reticulum stress was found to have the expression fold change ≥ 2.0, implying that long-term of 6-month metformin treatment has no significant impact on the “bad” genes in ZDF rat liver.

### Validation of target genes

**Fig. 4:**
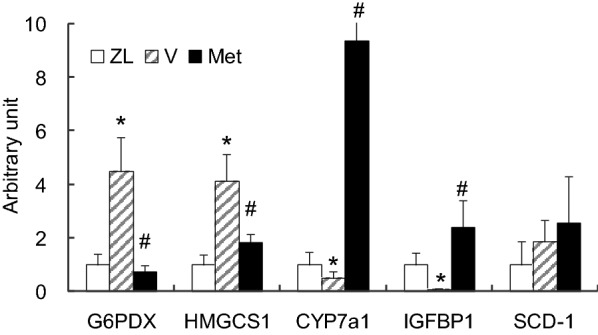
Real-time PCR quantification of target genes. Total mRNA of liver tissues were extracted from Zucker lean rats (ZL), vehicle-treated ZDF rats (V) and metformin-treated ZDF rats (Met) after 6-month treatment. Genes with interests were validated by quantitative real-time PCR. Similar to the results of microarray assay, gene expression levels of G6PDX, HMGCS1, CYP7a1 and IGFBP1were modified by leptin receptor mutation and corrected by metformin treatment, while the expression level of SCD-1 was not corrected by metformin treatment. Data are mean ± SD, *p < 0.05, V versus ZL; ^#^*p* < 0.05, Met versus V

Genes of interests were further validated by quantitative real-time PCR. We selected genes of the key enzymes involved in glucose (G6pdx) or lipid (Hmgcs1, Cyp7a1, Scd-1) metabolisms, and the gene with the most remarkable fold change (Igfbp1). Consistent with the microarray assay findings, gene expression levels of Igfbp1, Cyp7a1, Hmgcs1 and G6pdx were modified by the leptin receptor mutation and corrected by metformin treatment in the ZDF rats, whereas the expression level of Scd-1, an enzyme involved in fatty acid biosynthesis, was not corrected by the metformin treatment (Fig. 4).

## Discussion

In this study, we investigated the therapeutic potential of metformin, a widely applied insulin sensitizer, in treating NAFLD. We performed unbiased transcriptomic analysis (microarray) in ZDF insulin resistance and NAFLD rat model to compare gene expression changes between groups treated with vehicle and relatively chronic metformin. Our study showed that 6-month treatment with metformin significantly lowered post-load blood glucose levels without affecting blood lipid profiles or the hepatocyte degeneration process. Metformin treatment showed the trend of alleviating the increase of liver enzyme levels, but this alleviation did not reach the statistical difference. Above that, as ZDF rats develop insulin resistance at a relatively early age (6–8 weeks), and the metformin treatment was started at the age of 8 weeks, our study also suggested that early interference with insulin resistance using metformin does not seem to prevent the development of liver steatosis. Clinical trials are rare to study the efficacy of insulin sensitizer in changing the risk of developing obesity-associated NAFLD, our study thus offered unique preclinical data for answering this important question. Another unexpected finding of this study was that most of the genes corrected by the metformin treatment were those involved in the metabolisms of glucose and cholesterol, but not of fatty acids. As lipid (both fatty acids and triglycerides) accumulation in the liver is an important initiation step in the development of NAFLD, it is, therefore, consistent with our finding that metformin treatment did not improve the liver histology in the ZDF model. Our data also suggest that metformin improved cholesterol metabolism (based on the gene expression changes), which is a topic insufficiently explored. Whether metformin protects liver cholesterol dysregulation or cholestasis represents an extremely interesting question that remains to be answered.

One important question in the NAFLD field is how NAFLD progresses to more severe stages of non-alcoholic steatohepatitis (NASH) or fibrosis. It is currently unclear whether metformin helps to protect NAFLD from developing NASH or fibrosis. Based on our data, we found that no genes associated with inflammation, apoptosis, fibrosis, or cell death were normalized in the metformin-treated ZDF rats. These data suggest metformin does not protect NAFLD from developing into more severe NASH or fibrotic stages, which requires further investigation.

Clinically, several studies have shown diabetic patients with fatty liver disease had improved serum aminotransferases level and insulin resistance after metformin treatment [6, 10–12]. Therefore, metformin was used as a treatment candidate for NAFLD [3–6]. However, meta-analyses conclude that metformin therapy did not improve liver histology in patients with NAFLD or NASH [19, 20]. In the latest AASLD guideline for NAFLD treatment, metformin is not recommended for treating NASH in adult patients [21].

It is notable that many genes affected by administration of metformin were overcorrected after the treatment, which implied the variation levels of most corrected genes are much greater than those in Zucker lean rats. For example, IGFBP1, which participates in multiple metabolic processes including lipid and glucose metabolism, showed down-regulation with fold change of 5.3 in vehicle-treated ZDF rats compared with that in Zucker lean rats, whereas it was 19.3-fold higher in metformin-treated ZDF rats compared with vehicle-treated ZDF rats. Consistently, other important lipid- and glucose-related enzymes (such as Cyp7a1, Sqle, Sds) showed similar expression profiles. Overcorrection of the metabolic genes by metformin might contribute to more severe hepatocyte injuries.

CCO is a component in the mitochondrial respiratory electron transport chain and functions in the generation of ATP derived from protons mainly produced during glucose and lipid catabolic processes. In this study, we demonstrated the overexpression of CCO protein and some genes related to glucose and cholesterol catabolic processes in ZDF rats treated with either vehicle or metformin. This overexpression may reflect a hyperfunctional state of mitochondria to compensate for the dysfunction in degenerated cells. Stronger CCO staining at the edge of the vacuoles and AMPK activation in the degenerated hepatocytes were found in the metformin-treated rats, which may reflect the hyperfunctional status of mitochondria with the intention to consume the cellular lipids. However, it is unclear whether metabolic overburden aggravates the pathological changes of the hepatocytes.

It should be borne in mind that the observations from animal models cannot be completely extrapolated to humans. In the ZDF rat model, leptin receptor gene mutation is a complicated scenario leading to rather severe hyperlipidemia. Therefore, the degenerated hepatocytes may not be replaced by new generation of hepatocytes under the background of severe hyperlipidemia and gene deficiency. In humans, however, these abnormalities may not exist. Thereafter, the leptin gene deficiency and severe hyperlipidemia might be responsible for the ineffectiveness of the long-term metformin treatment on fatty liver in Zucker rats. In addition, the sample size of this study is relatively small, which might be a limitation to show a protective effect of metformin on liver enzymes. Further experiments and larger sample size can produce more reliable results.

## Data Availability

The datasets used and/or analyzed during the current study are available from the corresponding author on reasonable request.

## Abbreviations

ALT: alanine aminotransferase
AST: aspartate transaminase
CCO: cytochrome c oxidase
Cyp7a1: cytochrome P450, family 7, subfamily a, polypeptide 1
G6pdx: glucose-6-phosphate dehydrogenase
IGFBP1: insulin-like growth factor-binding protein-1
NAFLD: non-alcoholic fatty liver disease
NASH: non-alcoholic steatohepatitis
OGTT: oral glucose tolerance tests
ZDF: Zucker diabetic fatty
